# Amelogenesis Imperfecta and Generalized Gingival Overgrowth Resembling Hereditary Gingival Fibromatosis in Siblings: A Case Report

**DOI:** 10.1155/2012/428423

**Published:** 2012-10-09

**Authors:** Emre Yaprak, Meryem Gülce Subaşı, Mustafa Avunduk, Filiz Aykent

**Affiliations:** ^1^Department of Periodontology, Faculty of Dentistry, Kocaeli University, Kocaeli, Turkey; ^2^Department of Prosthodontics, Faculty of Dentistry, Aydın University, İzzettin Çalışlar Cad., No. 31/A, Bahçelievler, İstanbul, Turkey; ^3^Department of Pathology, Meram Faculty of Medicine, Necmettin Erbakan University, Konya, Turkey; ^4^Department of Prosthodontics, Faculty of Dentistry, Selcuk University, Konya, Turkey

## Abstract

Amelogenesis imperfecta (AI) is a group of hereditary disorders primarily characterized by developmental abnormalities in the quantity and/or quality of enamel. There are some reports suggesting an association between AI and generalized gingival enlargement. This paper describes the clinical findings and oral management of two siblings presenting both AI and hereditary gingival fibromatosis (HGF) like generalized gingival enlargements. The treatment of gingival enlargements by periodontal flap surgery was successful in the management of the physiologic gingival form for both patients in the 3-year follow-up period. Prosthetic treatment was also satisfactory for the older patient both aesthetically and functionally.

## 1. Introduction

Amelogenesis imperfecta (AI) is a group of hereditary disorders primarily characterized by developmental abnormalities in the quantity and/or quality of enamel, such as hypoplasia, hypomaturation, and/or hypocalcification [1, 2]. Dental anomalies such as multiple impacted teeth, failed tooth eruption, congenitally missing teeth, open occlusal relationship, taurodontism [3–8], pulpal calcification, hypercementosis, root or crown resorption, and root malformations [9–13] can be detected in AI patients.

Teeth attrition can be observed as a result of AI [14, 15]. Dentin hypersensitivity and decreased occlusal vertical dimension due to attrition causes a restrictive effect on teeth function and individual oral hygiene applications. Unaesthetic appearance is also disturbing factor for the AI patients, because of both color defects and shape abnormalities [16, 17]. Witkop's classification which, based on clinical and hereditary conditions, is the most frequently used among various classifications of AI [18–24]. 

Hereditary gingival fibromatosis (HGF) is a rare disorder characterized by the proliferate fibrous overgrowth of the gingival tissue that can occur as an isolated disease or as part of a syndrome or chromosomal abnormality [25, 26]. There are some reports suggesting an association between AI and gingival enlargement [12, 27–31]. However, the certain mechanism of the association of AI and gingival overgrowth remains in suspense.

This paper describes the rehabilitations of two female siblings presenting AI and HGF like generalized gingival enlargement.

## 2. Case Presentation

**Figure 1:**
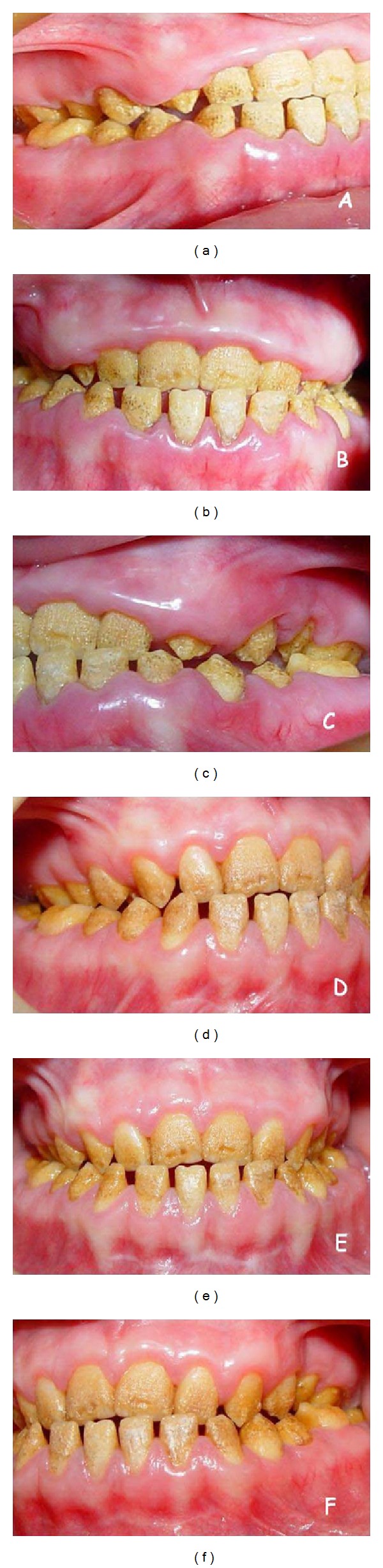
Initial aspect of the 12-year-old patient displays diffuse gingival enlargements covering almost all teeth ((a), (b), and (c)). Appropriate gingival form is established by periodontal flap surgery and maintained for 3 years ((d), (e), and (f)).

**Figure 2:**
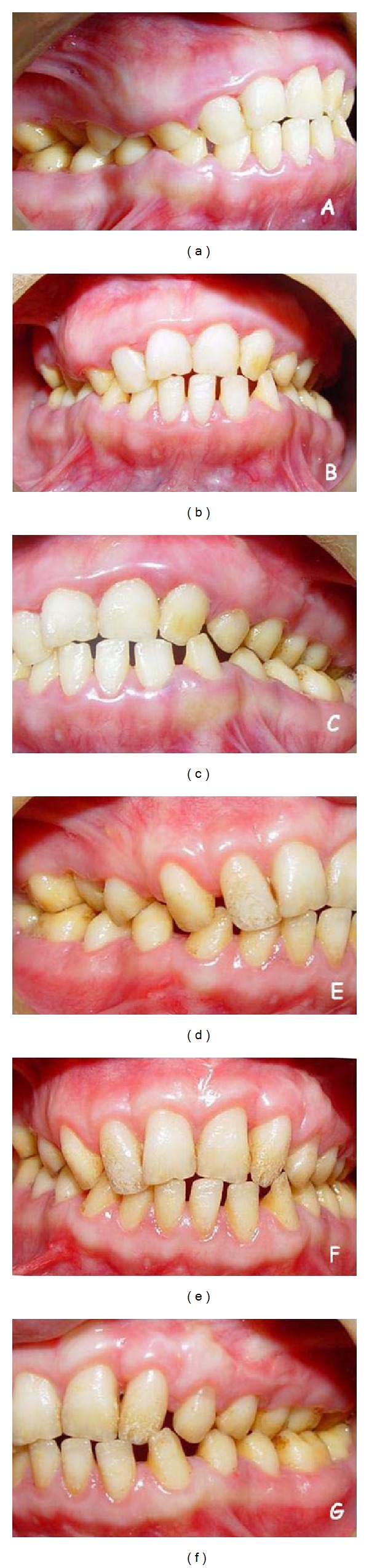
Initial appearance of 19-year-old patient presents more mild gingival enlargements comparing with her younger sibling ((a), (b), and (c)). Appropriate gingival form is constituted by periodontal flap surgery at the 3rd month before prosthetic treatment ((d), (e), and (f)).

**Figure 3:**
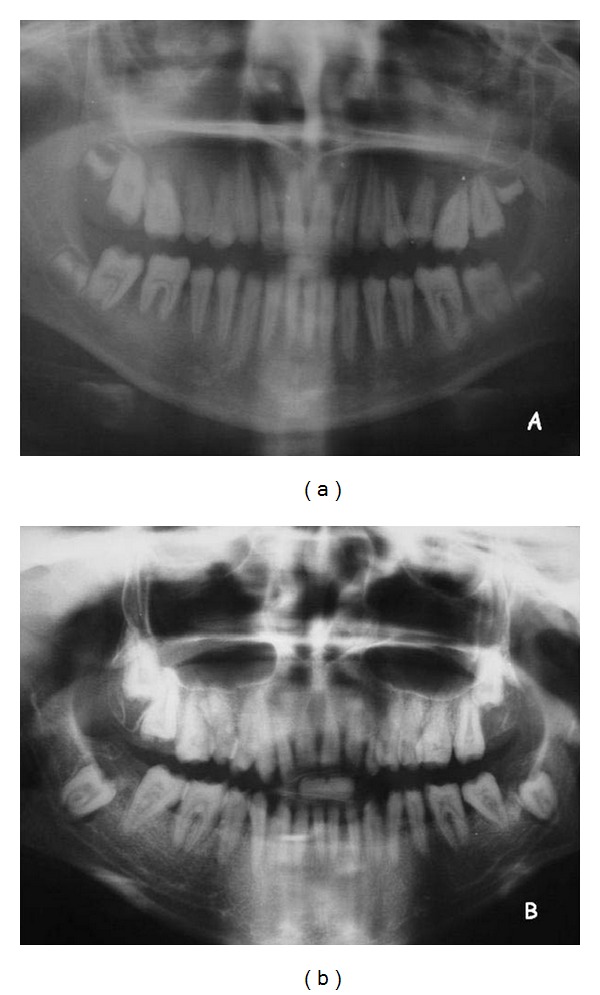
Panoramic radiography shows reduction of enamel thickness for younger (a) and older (b) patients.

Systemically healthy 12 and 19 years old female siblings, complaining of insufficient chewing functions due to hypersensitivity of the teeth and esthetic problems because of both gingival enlargement and the color of the teeth were admitted to the Periodontology Clinics at the Faculty of Dentistry, Selcuk University. Medical conditions of the patients were normal and there were no signs of any syndrome. Intraoral examination of both patients revealed yellowish-brown, rough, and atypical enamel formation. Generalized fibrotic gingival enlargements with secondary inflammation covering almost all teeth were also seen. Clinical crown lengths were short because of AI and diffuse gingival enlargement in both patients (Figures 1(a), 1(b), and 1(c)) and Figures 2(a), 2(b), and 2(c). However, these findings were more severe in the younger sister. O'Leary plaque index [32] was found 83% in younger and 71% in older patient. Radiographic examination showed normal dentin formation and pulp chamber, but enamel appeared to be thin in each patient (Figure 3). The other family members were examined except grand-parents and no familial occurrence of AI or gingival hyperplasia was observed.

**Figure 4:**
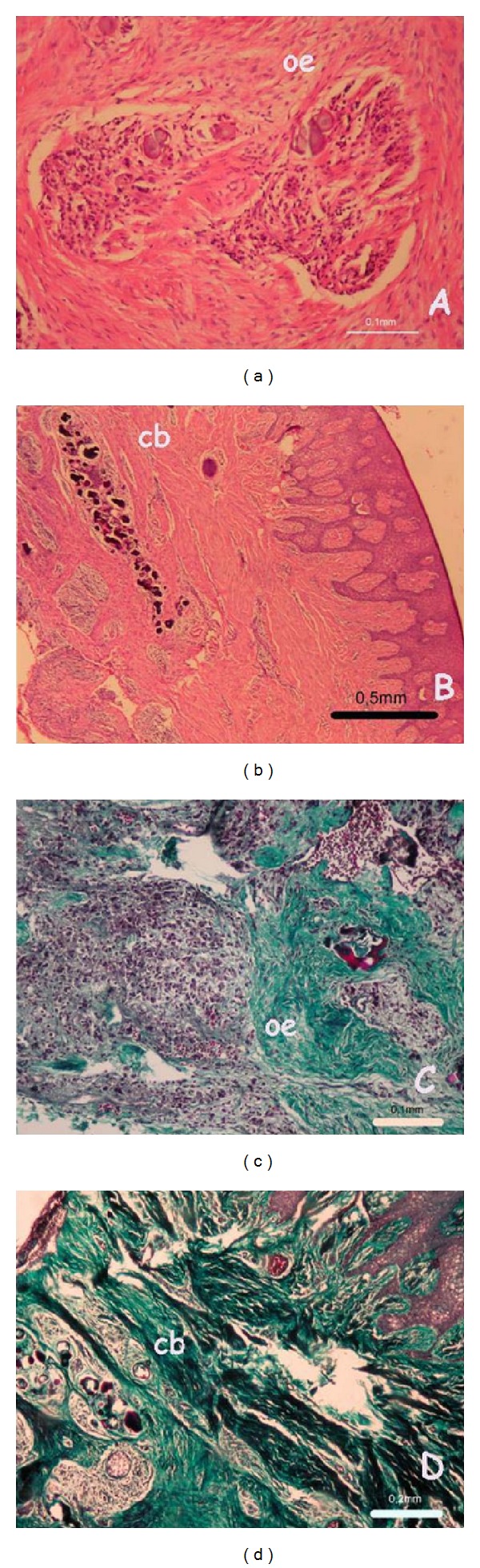
Odontogenic epithelium (oe), and calcified bodies (cb) were seen in hematoxylin and eosin ((a) and (b)) and Masson's trichrome ((c) and (d)) stainings of younger ((a) and (c)) and older ((b) and (d)) patients.

Clinical findings determined the diagnosis as a pitted hypoplastic type of amelogenesis imperfecta in younger and a rough hypoplastic type of amelogenesis imperfecta in older patient. Histological investigations showed mononuclear inflammatory infiltrate, calcified bodies, and odontogenic epithelium in a dens-fibrovascular-connective tissue (Figure 4). Because of fibrotic appearance, hereditary pattern, and histological character, the lesions were described as HGF like gingival enlargements with secondary inflammatory involvement for both patients.

**Figure 5:**
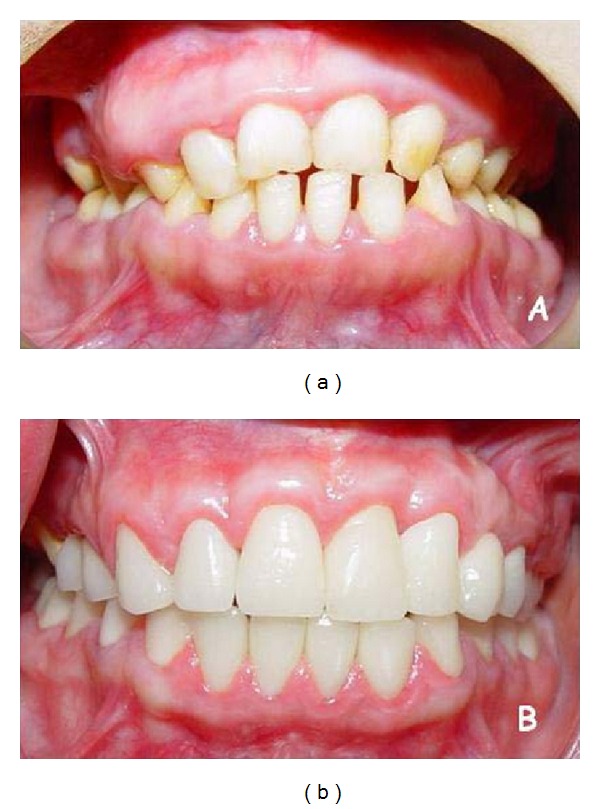
Preoperative (a) and the final (b) intra oral appearances of 19-year-old patient at the 3rd year.

The reduction of the gingival inflammation which was the main objective of the initial periodontal therapy was realized by oral hygiene motivation, scaling and root planning. Dental hypersensitivity limiting the plaque control was removed by fluoride gel applications and fluoride mouth washes. After obtaining an adequate hygiene and reduction of inflammation, excessive gingival tissues were excised by internal bevel incision with full-thickness flap elevation under local anesthesia. Maxillary and mandibular labial frenectomy and vestibuloplasty applications were done also during the surgery. In addition, some alveolar bone resection was applied to the right upper quadrant of the older patient. Thus, physiological gingival form and sufficient crown lengthening were constituted before the prosthetic treatment. In the follow-up period, at 3rd year, there was no sign for recurrence of gingival enlargement in both patients (Figures 1(d), 1(e), and 1(f) and Figure 5(b)).

After the periodontal therapy, two siblings were oriented to the department of prosthodontics. Clinical examination of younger patient revealed Angle Class III dental relationship, posterior cross-bite, pitted enamel defect, teeth attrition, and low vertical dimension. The older patient had Angle Class I dental relationship, posterior cross-bite, yellowish-brown teeth, rough enamel surface, teeth hypersensitivity, multiple diastema and normal occlusal vertical dimension. Radiographic examination of each patient, showed reduction of enamel. In the older patient as well as enamel wear maxillary right canine was congenitally missing. Both patients were diagnosed hypoplastic amelogenesis imperfecta. Since the younger patient did not complete developmental period, prosthodontic treatment was delayed. 

For the older patient, residual maxillary right primary canine was extracted, maxillary right lateral, mandibular left, and right first molar teeth received endodontic treatment. Full mouth all-ceramic fixed partial dentures (Zirconia FPDs) were planned to maintain more effective chewing function and also to obtain a better aesthetic appearance. Maxillary and mandibular anterior and posterior teeth were prepared for full ceramic restorations. To prevent the exposition of the pulp mandibular, four incisors were prepared creating a chamfer cervical finish line and the others were prepared with a rounded shoulder finish line. After completion of the preparations, gingival retraction was made and definitive impressions were made using a vinyl polysiloxane impression material (Elite H-D; Zhermack, Rovigo, Italy). Laboratory processed provisional restorations were cemented with noneugenol temporary cement (Temp Bond NE; Kerr, Salerno, Italy). The maxillary and mandibular casts with trimmed dies of prepared teeth were obtained from the type 4 dental stone (Elite Rock; Zhermack, Rovigo, Italy). Then the casts were mounted onto an articulator (Stratos 100; Ivoclar Vivadent, Schaan, Liechtenstein) using interocclusal records. Maxillary anterior right 4-unit zirconia FPD and 23 zirconia crowns were fabricated. The patient's natural occlusal scheme (right side group functional occlusion, left side canine protected occlusion) and anterior guidance were preserved in the definitive restorations. The crowns evaluated intraorally, adjusted, and cemented with a resin cement (Multilink; Ivoclar-Vivadent, Schaan, Liechtenstein). Postoperative care instructions were given to the patient on home care and periodic call visits. After the prosthetic treatment, the patient was satisfied both functionally and aesthetically (Figure 5(b)). Recall evaluations at 6-month intervals were performed for a period of 3 years. In the follow-up period, at 3rd year there was no sign for recurrence of gingival enlargement in both patients. 

## 3. Discussion

This paper described two female siblings presenting both AI and HGF like generalized gingival enlargements. The treatment of gingival overgrowth by periodontal flap surgery was successful in the management of the physiologic gingival form and the constitution of crown lengthening before prosthetic treatment. 

In the literature, there are case reports presenting an association between AI and gingival enlargement and most of these [12, 27–31, 33] reported a gingival enlargement accompanied with AI as an inflammatory gingival hyperplasia due to rough surface of the enamel increasing bacterial plaque accumulation. Histological findings similar to these reports such as mononuclear inflammatory infiltrate, calcified-bodies and odontogenic epithelium in a dens-fibrovascular-connective tissue, and ulceration of mucosa were also seen in current paper. Although Macedo et al. [33] considered these findings as occasional, these histological findings can be seen in HGF [34, 35].

This paper suggests an association between AI and HGF like overgrowth based on histological, clinical findings, and also hereditary pattern of siblings. A former report had a hypothesis that rough enamel surface due to AI increases bacterial plaque accumulation and this accumulation causes inflammatory gingival hyperplasia [33]. This consideration is fairly reasonable, but, however, there are many cases [16, 36, 37] of AI not presenting any gingival enlargements even inflammatory hyperplasia. So, whether there is another possible mechanism in the etiology of such generalized overgrowths in AI patients remains as a question. Beyond the separate histopathologic examinations, some case-control studies evaluating the role of fibroblastic activities, growth factors, are needed to determine the certain mechanisms of such gingival overgrowths with AI, and whether such enlargements resemble HGF or not.

AI has a large group of hereditary patterns such as autosomal dominant, autosomal recessive, sex-linked, and spodadic AI [1, 38–40]. HGF is also usually identified as an autosomal dominant condition although recessive forms are described in the literature [25, 35, 41, 42]. Some genetic linkage studies are needed to evaluate whether there is a genetic transition between AI and such gingival overgrowths or not.

Prosthetic rehabilitation is another important issue for the management of the aesthetical and functional complaints of AI patients. There are several prosthetic treatment alternatives for these patients. Porcelain fused to metal crowns, all ceramic crowns, laminate veneers, and overdentures can be used for the prosthetic treatment of AI patients [11, 17, 43]. The treatment plan is related to many factors such as number of permanent teeth, intraoral situation at the time of the treatment was planned, type and severity of disorder, patient's occlusion, occlusal vertical dimension, age, socioeconomic status of the patient, and patients expectation.

In this paper, considering these factors fixed full-mouth all ceramic prosthetic restorations were planned for the older patient. With the help of the prostheses adequate crown form, shape, and smooth crown surfaces were constituted and poor chewing functions were improved. Insufficient esthetic was also recovered by color management.

## 4. Conclusion

This paper describes clinical findings and oral rehabilitation of two female siblings presenting AI and HGF like generalized gingival enlargements. Periodontal flap surgery was successful in the treatment of gingival overgrowth of both patients. After the periodontal treatment of the older patient, full ceramic fixed restorations were effective to eliminate tooth hypersensitivity, improve esthetics, and restore function. The 3-year recall examination revealed no signs of deterioration in the restorations. The patient did not experience tooth sensitivity or any other complication associated with the oral rehabilitation. The patient's esthetic and functional expectations were also satisfied.
